# Personality Disorders and Post-Discharge Relapse in Mandated Inpatient Substance Use Treatment

**DOI:** 10.1192/bjo.2026.11225

**Published:** 2026-06-30

**Authors:** Faycal Ikhlef, Ahmad Alater, Zeeshan Sheikh, Majid Alabdulla, Nirvana Chandrappa

**Affiliations:** Hamad Medical Corporation, Doha, Qatar

## Abstract

**Aims:**

Personality disorders (PDs) are frequently assumed to confer poorer prognosis in substance use disorder (SUD) treatment and are often viewed as markers of higher clinical risk, reduced treatment responsiveness, and increased likelihood of relapse. These assumptions can influence clinical decision-making, expectations of recovery, and access to specialised services. However, empirical evidence supporting these beliefs remains limited and inconsistent, particularly within mandated rehabilitation systems where treatment engagement, supervision, and monitoring differ substantially from voluntary care. Data from the Middle East are especially sparse. This study aimed to examine the association between comorbid PD and post-discharge relapse following inpatient SUD treatment in Qatar and to characterise the prevalence of dual diagnosis within a mandated rehabilitation population. We hypothesised that comorbid PD would be associated with higher relapse rates following discharge.

**Methods:**

A retrospective cohort analysis was conducted using medical records of patients discharged from an inpatient SUD rehabilitation programme operating under a mandated treatment framework. The cohort comprised 72 patients who completed inpatient treatment and entered structured post-discharge monitoring. Patients were categorised according to the presence (n=19, 26.4%) or absence (n=53, 73.6%) of a diagnosed personality disorder documented during admission. Relapse was defined using clinically relevant real-world indicators, including documented positive urine drug screening, self-disclosed substance use during follow-up, or failure to attend scheduled drug testing. Relapse outcomes were compared between groups using chi-squared testing to assess the association between PD status and early post-discharge relapse.

**Results:**

Comorbid PD was identified in over one-quarter of the cohort, demonstrating a substantial burden of dual diagnosis within mandated rehabilitation settings. Relapse occurred in 36.8% of patients with comorbid PD (n=7) compared with 41.5% of patients without PD (n=22). The difference in relapse rates between groups was not statistically significant (χ²=0.0069; p=0.9336). Contrary to prevailing assumptions, relapse rates were numerically higher among patients without PD, although this difference was small and clinically modest. Overall, PD status did not meaningfully differentiate short-term relapse risk following discharge in this cohort.

**Conclusion:**

This study represents one of the first regional evaluations examining relapse outcomes by PD status within a mandated SUD rehabilitation context. Findings challenge assumptions that comorbid PD necessarily predicts poorer early recovery or higher relapse risk. Results support the feasibility of managing dual diagnosis patients within standard addiction treatment pathways when comprehensive assessment, structured monitoring, and integrated care are in place. Larger prospective studies are required to explore the impact of specific PD subtypes and longer-term outcomes.

